# Is the Timed Up and Go test a useful predictor of risk of falls in community dwelling older adults: a systematic review and meta- analysis

**DOI:** 10.1186/1471-2318-14-14

**Published:** 2014-02-01

**Authors:** Emma Barry, Rose Galvin, Claire Keogh, Frances Horgan, Tom Fahey

**Affiliations:** 1 HRB Centre for Primary Care research, Department of General Practice, Royal College of Surgeons in Ireland, 123 St. Stephens Green, Dublin 2, Republic of Ireland

## Abstract

**Background:**

The Timed Up and Go test (TUG) is a commonly used screening tool to assist clinicians to identify patients at risk of falling. The purpose of this systematic review and meta-analysis is to determine the overall predictive value of the TUG in community-dwelling older adults.

**Methods:**

A literature search was performed to identify all studies that validated the TUG test. The methodological quality of the selected studies was assessed using the QUADAS-2 tool, a validated tool for the quality assessment of diagnostic accuracy studies. A TUG score of ≥13.5 seconds was used to identify individuals at higher risk of falling. All included studies were combined using a bivariate random effects model to generate pooled estimates of sensitivity and specificity at ≥13.5 seconds. Heterogeneity was assessed using the variance of logit transformed sensitivity and specificity.

**Results:**

Twenty-five studies were included in the systematic review and 10 studies were included in meta-analysis. The TUG test was found to be more useful at ruling in rather than ruling out falls in individuals classified as high risk (>13.5 sec), with a higher pooled specificity (0.74, 95% CI 0.52-0.88) than sensitivity (0.31, 95% CI 0.13-0.57). Logistic regression analysis indicated that the TUG score is not a significant predictor of falls (OR = 1.01, 95% CI 1.00-1.02, p = 0.05).

**Conclusion:**

The Timed Up and Go test has limited ability to predict falls in community dwelling elderly and should not be used in isolation to identify individuals at high risk of falls in this setting.

## Background

Falls are a leading cause of injury and activity limitation in older adults and the adverse effects associated with falling result in significant personal, social and economic burden. Approximately 30% of community dwelling people aged 65 years and over will fall each year [1]. Falls account for 40% of all injury deaths and lead to 20-30% of mild to severe injuries ranging from soft tissue injuries to fractures in the elderly [2]. The causes of falling are multi-factorial and include extrinsic (environment-related), intrinsic (person-related) and behavioural (activity-related) factors. Gait instability has been identified as a relatively consistent risk factor for falls and the majority of screening programmes to identify those at risk of falls comprise an assessment of gait and balance [3,4]. There are a number of performance orientated mobility assessment tools that assess aspects of balance and gait involved in normal daily activities. These tools serve to identify patients at risk of falling however, the sensitivity and specificity of existing tools is low [5]. One such example is the STRATIFY clinical prediction rule (St. Thomas Risk Assessment Tool in Falling elderly inpatients), which consists of five items that address risk factors for falling including past history of falling, patient agitation, visual impairment affecting everyday function, need for frequent toileting, and transfer ability and mobility. The STRATIFY rule yields a possible score between 0 and 5 (each item scoring 1 if present or 0 if absent). A recent systematic review examined the predictive value of the rule in elderly inpatients at risk of falls and found that at a score ≥2 points, the STRATIFY rule had only limited predictive ability with moderate summary estimates of sensitivity (0.67, 95% CI 0.52 – 0.80) and specificity (0.57, 95% CI 0.45 – 0.69) [6].

The TUG test is another commonly used screening tool for falls risk in the inpatient and the community setting. The TUG (Timed Up and Go) test was developed in 1991 as a modified timed version of the Get up and Go test [7,8]. To perform the TUG test as described in the original derivation study, the patient is timed while they rise from an arm chair (approximate seat height 46 cm), walk at a comfortable and safe pace to a line on the floor three metres away, turn and walk back to the chair and sit down again. The subject walks through the test once before being timed to become familiar with the test. The subject wears his regular footwear and uses his customary walking aid (cane or walker) if necessary [8]. A faster time indicates a better functional performance and a score of ≥13.5 seconds is used as a cut-point to identify those at increased risk of falls in the community setting [9]. However, reported threshold values vary from 10 to 33 seconds in the literature [10,11].

The TUG is recommended as a routine screening test for falls in guidelines published by the American Geriatric Society and the British Geriatric Society [12]. The National Institute of Clinical Evidence (NICE) guidelines also advocate the use the TUG for assessment of gait and balance in the prevention of falls in older people [13]. To date three systematic reviews have examined the clinical utility of the TUG to discriminate between those at low and high risk of falling [14-16]. The most recent systematic review reported that the pooled mean difference in time taken to complete the TUG between fallers and non-fallers depended on the baseline functional status of the cohort of patients under investigation. In essence, there was a mean difference of 0.63 seconds (95% CI 0.14–1.12 seconds) in the performance of the TUG for high-functioning versus a difference of 3.59 seconds (95% CI = 2.18–4.99 seconds) for those in institutional settings [16]. The aim of this systematic review with meta-analysis is to examine the predictive value of the test to identify individuals at risk in falling in the community using the frequently cited cut-off of ≥13.5 seconds. A secondary aim of the study is to examine the summary estimates of sensitivity and specificity of alternative cut-off scores to optimally discriminate between fallers and non-fallers.

## Methods

### Search strategy

This systematic review and meta-analysis was performed according to the principles outlined by the Cochrane diagnostic test accuracy working group [17,18]. We aimed to identify all studies that validated the TUG test in community dwelling older adults. A systematic literature search was conducted in June 2012 (updated in March 2013) and included the following search engines: Pubmed, EMBASE, Cochrane Library, EBSCO, CINAHL and SCOPUS. A combination of the following keywords and MeSH terms were used: ‘Timed Up and Go test’, ‘Get Up and Go test’, ‘TUG’, ‘GUG’, ‘TGUG’, ‘TGUGT’, ‘ETGUG’, ‘ETGUGT’,’TUGT’, ‘modified TUG’ and ‘accidental falls’, ‘fall’, ‘falling’, ‘faller’. No language restrictions were applied to the search. The search was supplemented by hand searching reference lists of retrieved articles and searching Google scholar. The original version of the Get Up and Go test was created in 1986 [7], the timed version was later derived in 1991 [8], therefore only studies published from 1991–2013 were included in our literature review.

### Study selection and data extraction

Studies were included if they met the following inclusion criteria: 1) Prospective or retrospective cohort studies or randomised control trials, 2) Studies that included community dwelling older adults as the population of interest, 3) Studies that validated the original version of the TUG test, 4) Studies that recorded a subsequent fall. Studies were excluded if their population of interest was limited to patients with specific neurological or orthopaedic condition e.g. Parkinson’s disease, stroke, hip fracture or amputation of a lower limb. Studies were also excluded if they were limited to a population with a particular medical condition e.g. patients with chronic obstructive pulmonary disease. For the purposes of this review, we included studies where ≥80% of subjects were community dwelling and/or were described as self caring or independent. Studies where >20% of the subject population were described as institutionalised, living in nursing homes, residential care homes or geriatric inpatients were excluded. The definition of a subsequent fall was considered in the context of each individual study. We considered the following definition of a fall: ‘an unexpected event in which the patient comes to rest on the ground, floor or lower level as the reference standard [1] and variations of this definition were recorded in Table 1 that contains details of the included studies.

**Table 1:** Descriptive characteristics of the studies included in the review

**Study**	**Participants (n) sex, mean age (+/− SD)**	**Time frame of follow-up**	**Person administering test**	**Definition of falls**	**Method of administering the test**	**Method of recording falls**	**Number of individuals who fell**
Greene et al. 2012*	N = 349	2 years	Not recorded	Tinetti 1997	Test completed once. Assistive device not permitted.	Self report with collateral information from relatives and medical records	N = 83
	M = 103						
	F = 246						
	Mean age 71.5+/− 6.7						
Herman et al. 2011 and 2010	N = 265	3 years	Physiotherapist	Leveille 2009 and Mackensie 2006	Test performed twice (mean score used). Assistive device not permitted	Self report falls diary monthly	N = 64 (1 year follow up)
	M = 111						
	F = 154						N = 131 (2 year follow up)
	Mean age 76.4+/−4.3						
							N = 73 retrospective
Russell et al. 2008*	N = 344	1 year	Physiotherapist, occupational therapist or medical doctor	Kellogg 1987	As per original TUG.	Self report falls diary bi-monthly	N = 164
	M = 106						
	F = 238						
	Mean age 75.9+/−8.5						
Viccaro et al. 2011*	N = 457	1 year	Not recorded	Defined as unintentionally coming to rest on the ground or other surface.	As per original TUG. Average of 2 trials reported.	Interview at 3/12 visit	N = 174
	F = 201 M = 256						
	Mean age =74						
Killough et al. 2006*	N = 122	6 months	Unrecorded	Not defined	Administered test only once unless misunderstood and a second trial was used.	Interviewed every 3 months by telephone	N = not recorded
	M/F = unrecorded						
	Mean age = unrecorded						
Thomas unpublished study*	N = 31	12 months	Physiotherapist	Kellogg 1987	Shoe should have maximum heel height of 4 cm. TUG not described in any further detail.	Self report by monthly prepaid postcards and follow up phone call at 3, 6, 9/12.	N = 16 had 2 or more falls, 15 had 1 or no falls
	M = 6						
	F = 25						
	Mean age = 81.6						
Aoyama et al. 2011*	N = 58	6 months	Physiotherapist	Tinetti 1988	As per original TUG. Two test trials and mean score recorded.	Self report by falls diary, collected at 6 month	N = 25
	F = 58						
	M = 0						
	Mean age 80.5+/−5.7						
Sai et al. 2009*	N = 137	12 months	Trained clinical staff	Buchner 1993	Time taken for subject to get up from chair (with arms crossed across chest) walk 10 ft, turn around and sit back down as quickly as possible.	Self report by falls diary. This was followed by monthly phone-calls.	N = 70
	M = 48						
	F = 89						
	Mean age 76.7 +/− 6.1						
Alexandre et al. 2012*	N = 63	12 months	Trained Physical therapists	Kellogg Working Group 1987	As per original TUG seat height 42 cm, back 79 cm, arms 60 cm from ground. Participants used own foot wear and used assistive device if needed.	Interviewed every 3/12 by blinded evaluator and self report via log book collected every 3/12.	N = 21
	Male = 30						
	Female = 33						
	Mean age Fallers = 66.68+/−5.57.Non fallers =66.36+/−4.60						
Yamada et al. 2010	N = 171	1 year	Trained staff members	Koski 1996	As per original TUG, height 40 cm, 3 m at normal pace turn walk back to chair and sit down. 2 trials average time recorded	Monthly telephone calls using structured questionnaire. Self report by mail every month.	N = 59
	F = 134						
	M = 37						
	Age 80.5+/ -5.6						
Yamada et al. 2012	N = 252.	1 year	Trained researchers	Koski 1996	Participants asked to stand up from a standard chair seat height 40 cm, walk a distance of 3 m at **a maximum pace,** turn walk back and sit down. Better performance of two attempts recorded. Walking aid permitted	Interview at end of follow up	N = 71
	231 who completed study:						
	Male = 54						
	Female = 177						
	Mean age =						
	T1 = 73.9+/−6.6						
	T2 = 79.1+/−7.0						
	T3 = 82.0+/−6.9						
Wrisley et al. 2010	N = 35	6 months	Physical therapist	Defined as unintentional contact below pt’s height and classified as unexplained or unexplained. A fall was considered explained if there was medical environmental or task-related explanation for the fall that was unavoidable. An unexplainable fall was all other falls.	As per original TUG. Participants were allowed 1 practice trial and then preformed 3 timed trials. The average of 3 trials reported	Self report by daily falls calendar; return a separate postcard providing details of any falls with follow up phone call.	N = 17 (6 participants reported 7 unexplained falls)
	Mean age =72.9 +/−7.8						
	M = 17						
	F = 18						
Pai et al. 2010	N = 13	1 year	Not recorded	Defined as any event in which they landed unintentionally on a lower surface such as a chair, the floor or ground	As per original TUG, one practice trial given.	Contacted by telephone between 29–32 months into study	N = 4
	M = 9						
	F = 4						
	Mean age =72+/−5						
Okumiya et al. 1998	N = 328	5 years prospective	Not recorded	Not defined	As per original TUG	Self-administered questionnaire	68
	M = 151						
	F = 177						
	Mean age =80.3						
Lin et al. 2004	N = 1200	1 year	Trained interviewer	Not defined	As per original TUG standard chair with seat height of 40-50 cm height	Self report by postcard when a fall occurred and telephoned every 3/12.	Not recorded
	M = 709						
	F = 491						
	Mean age = 73.4						
Buatois et al. 2006	N = 206	16 months	Not recorded	Tinetti 1988	As per original TUG	Self report by falls calendar and questionnaire	N = 57
	M = 116						
	F = 90						
	Mean age = 70+/−4						
Buatois et al. 2010	N = 1618	Mean time 25+/−5 months (18–36 months)	Not recorded	Tinetti 1999	As per original TUG	Self report by questionnaire at end of study. Mean follow up period 25 +/−5 months range 18-36months.	N = 333
	M = 821						
	F = 797						
	Mean age 70						
Bergland et al. 2003*	N = 307	1 year	Not recorded	Falls defined as an unintentional change in position resulting in the victim lying on the floor or on the ground.	The subject was instructed to rise from a chair, walk 3 m as **quickly as possible,** cross a line, turn, walk back, and sit down again.	Self report by daily calendar, to return calendar every 3/12 with follow up phone call if a fall occurred. Subjects who did not return the calendar were contacted	155
					Wearing ordinary shoes and used customary walking aids if needed.		
	M = 0						
	F = 307						
	Mean age 80.8						
Trueblood et al. 2001	N = 198	6 months	Researcher	Anacker 1992	As per TUG, armless chair, 3 timed trials average time recorded	Telephone survey 4 and 6 months using a formalised script.	N = 30
	M = 38						
	F = 160						
	Mean age 78.1+/−8.2						
Shimada et al. 2009	N = 445	1 year	Day centre staff nursing allied health or similar qualifications	Nevitt 1989 Cumming 2008	As per TUG, measured once at usual pace assistive device allowed	Self report questionnaire, with collateral if difficulty in recall	N = 99
	F = 310						
	M =135						
	Mean age 80.5+/−7.2						
Melzer et al. 2009*	N = 100	1 year prospective	Research assistant	Tinetti 1988	Not described in detail but referenced as per AGS/BGS which in turn references Podsiadlo	Self report by daily calendar. Contacted by research assistant at one month intervals to monitor falls.	N = 49
	Male = 26						
	Female = 72						
	Mean age 78.4+/−5.7						
Beauchet et al. 2007 and 2008	N = 187	1 year	Trained evaluator	Defined as unintentionally coming to rest on the ground, floor or lower level	Not described reference to Podsiadlo	Monthly phone call using a standardised questionnaire. Collateral obtained if cognitive impairment	N = 54
	M = 29						
	F = 158						
	Mean Age = 84.8+/−5.2						
Garber et al. 2010	N = 904	6 months	Trained bilingual field interviewers	Not defined	Armless chair 3 m **as quickly** 2 trials –first practice second recorded time	Not recorded	Not recorded
	Male = 263						
	Female = 641						
	Mean age 76.6+/−0.5						

Asterix * indicates studies included in meta analysis.

Definition of a fall:

• Tinetti 1988 [1] “an event which results in a person coming to rest unintentionally on the ground or lower level, not as a result of a major intrinsic event (such as a stroke) or overwhelming hazard. An overwhelming hazard was defined as a hazard that results in a fall by the youngest healthiest person”.

• Levielle 2010 [53] “A fall was defined as unintentionally coming to rest on the ground or other lower level not as a result of a major intrinsic event (eg, myocardial infarction, stroke, or seizure) or an overwhelming external hazard (eg, hit by a vehicle).

• Mackenzie 2006 [54] “a fall was defined as an unintentional event where a person fell to the ground”

• Buchner 1993 [55] “unintentionally coming to rest on the ground, floor or some other lower level”.

• Kellogg Working Group 1987 [56]: “unintentionally coming to the ground or some lower level and other than as a consequence of sustaining a violent blow, loss of consciousness, sudden onset of paralysis as in stroke or epileptic seizure”.

• Anacher 1992 [57] “any disturbance of balance during routine activities that resulted in a person’s trunk, knee or hand unintentionally coming to rest on the ground or some level below the waist”.

• Cumming 2008 [58] “unintentionally coming to rest on the ground or other lower level not as a result of a major internal (for example, stroke) or external event”.

• Koski 1996 [59] “as an unexpected event where a person falls to the ground from an upper level or the same level”.

• Original TUG: Podsiadlo 1991 [8] “The timed up and go measures, in seconds, the time taken by an individual to stand up from a standard arm chair (approximate seat height of 46 cm), walk a distance of 3 metres, turn, walk back to the chair and sit down again. The subject wears his regular footwear and uses his customary walking aid (none, cane, or walker). No physical assistance is given. He starts with his back against the chair, his arms resting on the chair’s arms and his walking aid at hand. He is instructed that, on the word “go”, he is to get up and walk at a comfortable and safe pace to a line on the floor 3 metres away, turn, return to the chair and sit down again. The subject walks through the test once before being timed in order to become familiar with the test.

Two reviewers (EB, RG) read the titles and/or abstracts of the identified references and eliminated irrelevant studies. Studies that were considered eligible for inclusion were read fully in duplicate and their suitability for inclusion was independently determined by both RG and EB. Disagreement was managed by consensus. Data were extracted on study type and setting, patient demographics (age, gender) and clinical characteristics including relevant inclusion and exclusion criteria, person who administered the TUG, person who recorded the subsequent fall, the definition of a fall used. For the purposes of this paper, the unit of analysis was the patient or “faller” rather than each “fall” to avoid duplication bias. Authors were contacted by email to provide further information on patient cohorts where there was insufficient data provided. Studies that included data on the same patient cohort for more than one publication were only included once in the meta-analysis.

### Quality assessment

The methodological quality of the selected studies was evaluated independently by two reviewers (EB and FH) using the QUADAS-2 tool, a validated tool for the quality assessment of diagnostic accuracy studies [17,18]. This checklist consists of four key domains: patient selection, index test, reference standard and flow and timing. Within each study, the domains are assessed in terms of risk of bias and the first three of these domains are assessed in terms of concerns about applicability. Signalling questions as specified in the QUADAS-2 tool enable the reviewer to give each domain a rating of high, low or unclear. Disagreements were resolved by a third reviewer (RG).

### Statistical methods

We used Stata version 12 (StataCorp College Station, Texas, USA), particularly the metandi command that fits the bivariate model, for all statistical analyses. We have applied this methodology in similar studies [19]. Significance was set at p < 0.05 for all analyses. A 2 × 2 table was constructed to extract the number of true positives, false positives, true negatives, false negatives, for the TUG test from each validation study using the pre-defined cut-point of ≥13.5 seconds to identify those at increased risk of falling. We applied the bivariate random effects model to estimate summary estimates of sensitivity and specificity and their corresponding 95% confidence intervals. This approach preserves the two-dimensional nature of the original data and takes into account both study size and the heterogeneity beyond chance between studies [20]. Sensitivity refers to the proportion of fallers correctly classified as high risk. Specificity is the proportion of non-fallers correctly classified as low risk.

The sensitivity and specificity for the TUG test was plotted in a hierarchical summary receiver operating characteristic (HSROC) graph, plotting sensitivity (true positive) on the y axis against 1-specificity (false negative) on the x axis. The 95% confidence region and the 95% prediction region were plotted around pooled estimates to illustrate the precision with which the pooled values were estimated (confidence ellipse around the mean value) and to illustrate the amount of between study variation (prediction ellipse).

Heterogeneity was evaluated visually using the summary ROC plots and statistically by using the variance of logit transformed sensitivity and specificity, with smaller values indicating less heterogeneity between studies. Bayes’ theorem was used to estimate the post test probability of a fall by multiplying the pre-test odds by the likelihood ratio; where pre-test odds are calculated by dividing the pre-test probability by (1+ pre-test probability) and the post -test probability equals post test odds divided by (1 + post-test odds). We completed sensitivity analyses to explore the effect of methodological features, as determined by the QUADAS-2 tool, on the predictive value of the TUG test.

The *c* statistic, or area under the curve, with 95% CI were also estimated to describe model discrimination. The *c* statistic ranges from 0.5 (no discrimination) to a theoretical maximum of 1, values between 0.7 and 0.9 represent moderate accuracy and greater than 0.9 represents high accuracy. A c statistic of 1 represents perfect discrimination, whereby scores for all cases (fallers) are higher than those for all the non-cases (non-fallers) with no overlap [21]. Finally, the association between the TUG score and falls was assessed using logistic regression and is presented as odds ratios with 95% confidence intervals.

## Results

### Study identification

**Figure 1:**
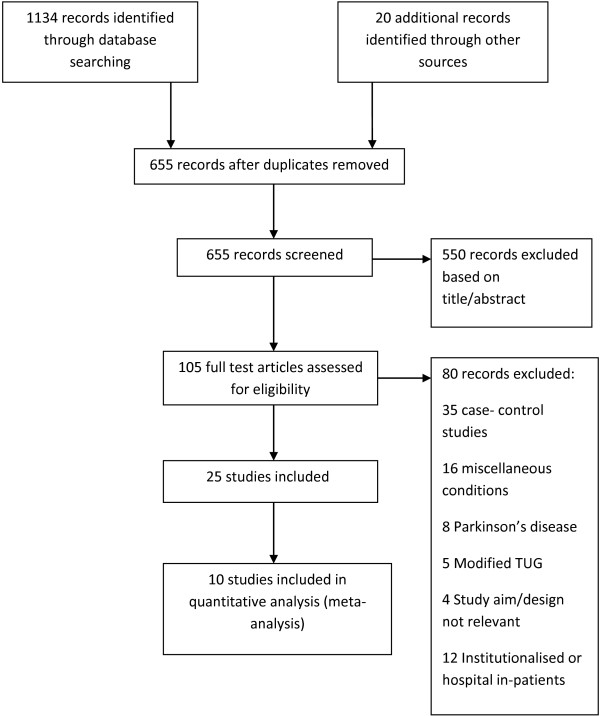
Flow diagram.

A flow diagram of the search strategy is presented in Figure 1. Two researches (EB, RG) screened all potential papers. The search strategy yielded 1,134 articles and an additional 20 articles were found by hand searching resulting in 1,154 articles. Six hundred and fifty five articles remained after duplicates were removed. Five hundred and fifty were then excluded based on title or abstract. Of the remaining 105 articles, 80 were excluded after reading the full text leaving 25 articles. Within this group, there were four publications based on two unique cohorts of patients [22-25].

### Study characteristics

The characteristics of the 25 prospective cohort studies are contained in Table 1. The descriptive characteristics were combined where two studies were based on the same population of patients [22-25]. In relation to 25 studies: seven studies were based in the USA [26-32], five in Japan [33-37], three in Israel [22,23,38], four in France [24,25,39,40] and one in each of Taiwan [41], Australia [42], the UK (unpublished) [43], Brazil [44], Ireland [45] and Norway [46]. The size of the patient cohort in the included studies ranged from 13 [29] to 1618 patients [40]. In total 2,314 patients were included in the meta-analysis from 10 different datasets. The duration of follow up varied from six months [27,28,31,32,34] to five years [33].

The application and the conditions of testing varied in many of the validation studies – variations included the instruction to walk as quickly as possible during the task [26,46], the sole use or non-use of an assistive device [36,45] standing from an armless chair [27], seat height variations 40 cm [35] to 50 cm [41], walking with arms crossed [26]. Testing conditions also varied in that some studies to allow a practice attempt and/or record the average time of two or three attempts [28].

### Study quality

**Figure 2:**
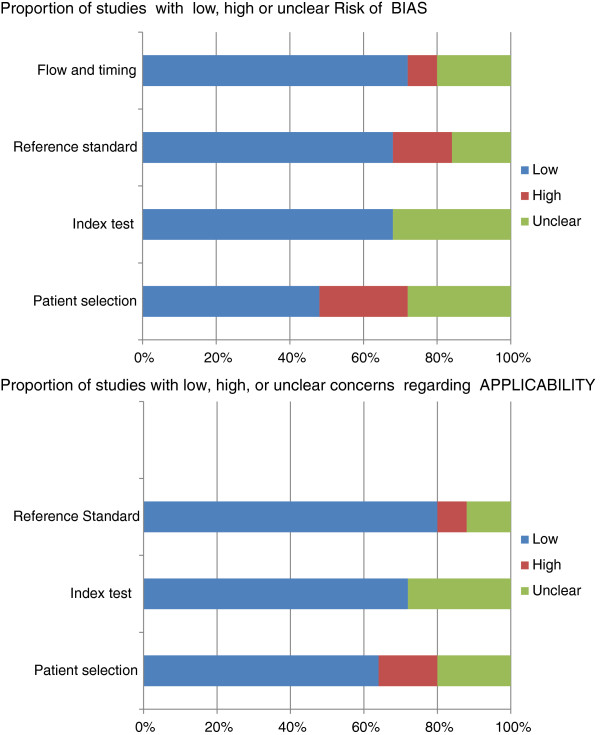
Methodological quality of the studies included in the review.

The summary diagram of the quality assessment is shown in Figure 2. All twenty five articles were quality assessed. The overall quality of the studies included was moderate with six studies [23,27,30,35,36,39] rated as low in all domains in both risk of bias and concerns about applicability. Ten studies [22,24-26,31,33,37,38,41,43] rated as having an unclear risk of bias and nine studies [28,29,32,34,40,42,44-46] were rated as having a high risk of bias. This was primarily attributed to a lack of information provided with respect to methods of patient recruitment (selection bias) and criteria used to ascertain of a subsequent fall (reference standard).

In relation to concerns about applicability of each individual study to the proposed research question, ten studies [23,26,27,30,35,36,39,40],[42,45] were rated as low, ten studies [22,24,25,29,33,37,38,41],[43,44] were rated as unclear and five studies [28,31,32,34,46] were considered as having high level of concern. A high or unclear risk of bias was noted in studies that inadequately described loss to follow-up in the cohort or the methods used record the incidence of a fall over the period of study. The index test was adequately described in the majority of studies but the many studies failed to record whether the index test (TUG score) was interpreted with or without knowledge of the reference standard (subsequent fall).

### Predictive accuracy of all included studies

**Figure 3:**
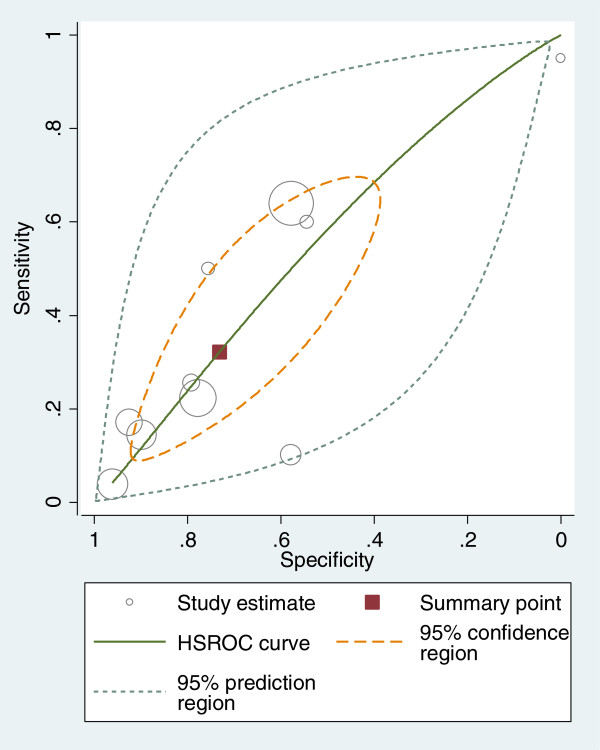
Hierarchical summary receiver operating characteristic plot of sensitivity and specificity for the TUG predicting falls at a cut point ≥13.5 seconds.

All authors were contacted to request primary data and ten authors responded with the relevant data [26,30,31,34,38,42-46]. In two of the ten studies where data was provided [26,46], the TUG was administered as quickly as possible and in the remaining eight studies it was administered at a comfortable pace. The duration of follow-up in these studies varied from six months [31,34] to two years [45]. The remaining seven studies followed patients for one year after administration of TUG [26,30,38,42-44,46]. The pooled sensitivity, specificity and the respective variance of the logit transformed sensitivity and specificity for the ten studies included in the meta-analysis are displayed in Table 2. These findings indicate that the TUG test is more useful at ruling in rather than ruling out falls in individuals classified as high risk (≥13.5 seconds), with a higher pooled specificity (0.73, 95% CI 0.51-0.88) than sensitivity (0.32, 95% CI 0.14-0.57). Individual and summary estimates of sensitivity and specificity for all studies, the 95% confidence region and 95% prediction region are presented in the summary ROC graph (Figure 3). The 95% confidence region is broad, reducing the precision of studies in the pooled estimate. The 95% prediction region (amount of variation between studies) is also wide suggesting heterogeneity between studies.

**Table 2:** Summary estimates of sensitivity, specificity, and positive and negative likelihood ratios for all included studies and for sensitivity analyses at a cut point of ≥13.5 seconds

**Application of TUG test**	**No. of studies (patients)**	**Sensitivity (95% CI)**	**Variance logit sensitivity (95% CI)**	**Specificity (95% CI)**	**Variance logit specificity (95% CI)**
**All studies**	10 (n = 2,314)	0.32 (0.14-0.57)	2.62 (0.94-7.29)	0.73 (0.51-0.88)	2.24 (0.76-6.63)
**Studies where TUG was administered as fast as possible excluded**	8 (n = 1,872)	0.44 (0.20-0.71)	2.52 (0.78-8.1)	0.71 (0.49-0.86)	1.7 (0.52-5.56)
**Studies with duration of follow up > or < one year excluded**	7 (n = 1,858)	0.33 (0.11-0.68)	3.58 (1.07-12.0)	0.70 (0.37-0.90)	3.33 (0.89-12.49)
**Studies with selection bias excluded**	6 (n = 1,253)	0.29 (0.10-0.60)	2.31 (0.56-9.56)	0.64 (0.20-0.93)	5.11 (0.86-30.47)
**Studies with unclear/no details on index test excluded**	6 (n = 1,636)	0.33 (0.17-0.54)	1.05 (0.30-3.63)	0.71 (0.58-0.81)	0.46 (0.12-1.65)
**Studies with unclear/no definition ‘fall’ excluded**	6 (n = 1,750)	0.28 (0.11-0.54)	1.82 (0.55-6.05)	0.81 (0.64-0.91)	1.10 (0.32-3.73)

**Figure 4:**
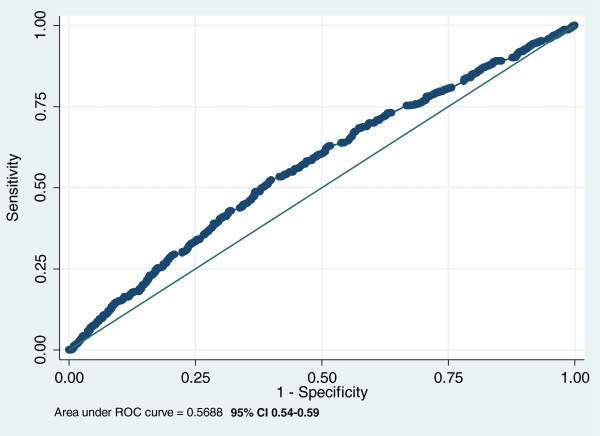
Performance of TUG to distinguish fallers from non-fallers.

The logistic regression analysis also indicates that the TUG score is not a significant predictor of falls (OR = 1.01, 95% CI 1.00-1.02, p = 0.04). The limited discriminative performance of the TUG is confirmed by the ROC curve analysis (Figure 4) indicating about 57% overall accuracy by a significant area under the curve (AUC_ROC_ = 0.57, 95% CI 0.54-0.59).

### Sensitivity analysis

A sensitivity analysis was completed excluding the two studies where the TUG was administered as fast as possible.[26,46] The summary estimates of sensitivity (0.44, 95% CI 0.20-0.71) and specificity (0.71, 95% CI 0.49-0.86) were broadly unchanged. The three studies where the duration of follow up was less than or greater than one year were removed [31,34,45]. Similarly, the summary estimates of sensitivity (0.33, 95% CI 0.11-0.68) and specificity (0.70, 95% CI 0.37-0.90) were unchanged. We also excluded four studies where there was evidence of selection bias [34,42,44,46]. Removal of these studies from the meta-analysis reduced the precision of the estimates of sensitivity (0.29, 95% CI 0.10-0.60) and specificity (0.64, 95% CI 0.20-0.93). Four studies [31,38,43,46] that did not adequately describe the method of administration of the TUG test were removed from the sensitivity analysis and the summary estimates of sensitivity (0.33, 95% CI 0.17-0.54) and specificity (0.71, 95% CI 0.58-0.81) of the TUG was broadly similar to the overall analysis. Finally we excluded four studies [26,31,43,45] where no clear definition of a fall was reported. While the sensitivity remained stable (0.28, 95% CI 0.11-0.54), the predictive ability of the TUG to rule in individuals at high risk of falling increased to 81% (95% CI 0.64-0.91). The pooled sensitivity, specificity and the respective variance of the logit transformed sensitivity and specificity for the studies included in the sensitivity analysis are displayed in Table 2.

### Bayesian analysis

Using Bayes’ theorem, the post-test probability of a fall across the different subgroups is presented in Table 3. The pre-test probability (prevalence) was calculated as 51% across all studies. The cut-point of ≥13.5 seconds has little impact on identifying those at high risk of falls when all studies are combined and across all of the different subgroups. Of note, when studies that provided no/unclear definitions of falls were excluded, the positive likelihood ratio increased to 1.50 (95% CI 1.15-1.94) and the post-test probability of a fall in patients classified as high risk increased from 54% to 64%.

**Table 3:** Post-test probability of a fall in patients classified as high risk (≥13.5 seconds) and low risk (<13.5 seconds) using the TUG score

**Application of TUG test**	**Pre test probability (%)**	**+ LR (95% CI)**	**Post test probability (%) + LR**	**-LR (95% CI)**	**Post test probability (%) -LR**
**All studies**	51% (49%-53%)	1.20 (0.82-1.75)	56% (54%-58%)	0.93 (0.78-1.10)	49% (47%-51%)
**Studies where TUG was administered as fast as possible excluded**	52% (49%-54%)	1.53 (1.31-1.79)	62% (59%-64%)	0.79 (0.62-1.01)	46% (44%-48%)
**Studies with duration of follow up > or < one year excluded**	53% (51%-56%)	1.11 (0.64-1.92)	56% (54%-59%)	0.95 (0.73-1.24)	52% (50%-55%)
**Studies with selection bias excluded**	52% (50%-55%)	0.81 (0.40-1.16)	47% (45%-50%)	1.10 (0.72-1.71)	54% (52%-57%)
**Studies with unclear/no details on index test excluded**	56% (54%-58%)	1.14 (0.67-1.91)	59% (57%-61%)	0.95 (0.74-1.21)	55% (53%-57%)
**Studies with unclear/no definition ‘fall’ excluded**	54% (51%-56%)	1.50 (1.15-1.94)	64% (61%-66%)	0.89 (0.75-1.04)	51% (48%-53%)

## Discussion

### Statement of principal findings

This systematic review demonstrates that the diagnostic accuracy of the Timed Up and Go test is limited at the widely used cut point of ≥13.5 seconds and should not be used for identifying community dwelling adults at high risk of falls in clinical practice. The sensitivity analysis which examined the performance of the rule in different subgroups also showed broadly comparable results, indicating that the TUG performed in a similar manner regardless of the methodological quality of the studies.

### Results in the context of the current literature

The TUG is commonly used in the research and clinical setting to screen individuals at increased risk of falling. The commonly cited cut-off score of ≥13.5 seconds used to identify individuals at high risk of falls was first described by Shumway-Cook and colleagues in 2000 [47]. However, the nature of the study design (case–control) used to derive the TUG was not optimal and subject to bias in terms of choosing appropriate controls and determining exposure. In addition, the study comprised of small numbers of patients with 15 fallers and 15 non-fallers included in the analysis. The definition of a fall was broad “any unplanned unexpected contact with a supporting surface, excluding unavoidable environmental hazards” and the study excluded those who had had one or fewer falls in the previous six months. The authors reported a sensitivity of 80% and specificity of 100%, suggesting that the TUG is more useful at ‘ruling-in’ falls in those classified as high risk. However, these findings need to be interpreted in the context of the methodological limitations of the study.

This systematic review only included cohort studies and randomised controlled trials where the index test (TUG) preceded the outcome of interest (fall) and the findings are in keeping with those reported in a previous systematic review that included both case–control and cohort studies [16]. The authors reported that the predictive accuracy of the TUG in identifying fallers across the included studies was poor to moderate and sensitivity and specificity were often close to chance [16]. Furthermore, cut-off points for identifying patients at increased risk of falls in independent-living patients varied between 8.1-16 seconds for performing TUG at a comfortable speed and between 11–13.5 seconds at a fast walking speed.

The limited predictive value of the TUG may be explained by the fact that the TUG is a single test which reflects strength balance and mobility nonetheless, the risk of falling has been shown to depend on multiple intrinsic and extrinsic factors [48,49]. The TUG does not appear to adequately encompass these risk factors. Recent literature has focused on the addition of a second manual [50] or cognitive task [51]. Nevertheless, the predictive ability of the tool remains limited with the inclusion of these tasks. Further study of the constituent parts of the TUG by quantifying body movements through the use of body worn sensors have increased the predictive accuracy of the TUG to almost 80% in one study [45].

### Strengths and weaknesses of the study

This study pooled data from a broad range of studies enhancing the generalisability of its findings. We examined the methodological quality of the studies using the validated QUADAS-2 tool for assessing the quality of such studies. In addition, sensitivity analyses examined the effect of important methodological variables including studies with selection bias, index test bias and reference standard bias. We also used individual patient data rather than aggregate data to calculate summary estimates of sensitivity and specificity and their corresponding 95% confidence intervals. This allowed more accurate data analysis by accounting for heterogeneity between studies and influences of sample size. However, the findings from the systematic review need to be interpreted in the context of the study limitations. Significant heterogeneity exists between the validation studies with respect to variation in the application of the TUG and a lack of information relating to the conditions of performing the TUG e.g. shoes worn, floor surface, chair seat and arm height, walk to a line or an X on floor. Studies have shown that these factors can affect TUG performance, time to complete the test was found to be significantly longer when a chair without armrests was used [52]. Studies varied in the number of practice trials given and an average result recorded. In other studies up to three attempts were given before a timed trial was done. Furthermore, some studies did not allow the use of assistive walking device and others specified the flooring type which has also been shown to affect TUG score.

### Clinical implications

Falls risk screening tools are an important element of falls prevention in the community. It is necessary to identify patients at high risk for falls and to facilitate the effective delivery of appropriate interventions to such patients. Inaccuracy of falls screening tools leads to inappropriate distribution of resources, contributing to varying degrees of success and failure of falls prevention strategies. It is essential to establish the accuracy of such tools and identify alternative tools that may be able to identify patients at risk of falling more accurately. Despite a growing body of evidence indicating its limited ability to predict falls, the TUG continues to be mentioned in clinical guidelines as a potential tool to identify fallers [12,13]. This is most likely because it is easy and quick to perform and does not require specialist equipment. Nonetheless, the totality of evidence to date is that it has limited predictive ability and should not be used in isolation to identify community-dwelling older people at increased risk of falls. Clinicians who assess the elderly for risk of falling should ideally do so in a comprehensive manner, taking into account the multi-factorial nature of falls rather than relying on a single test of mobility.

### Areas of future research

This study demonstrates that the TUG should no longer be used as a falls risk assessment in community dwelling elderly people. Gait, balance and to a lesser degree vision and cognition are inherently assessed in the TUG however, it does not include other accepted risk factors for falls e.g. medication use and morbidity. Further research is needed to determine its usefulness in lower functioning groups and those who have specific deficits in the areas of balance and mobility.

Advancing age is a primary risk factor for falling and recent studies have shown that the rate of falling remains at approx 30% in older people since 1988 [2]. The reasons why the elderly fall continues to be explored in the literature, and further research is required to develop a comprehensive falls risk tool that can accurately identify the common risk factors that predict falls. In order to prevent falls and reduce the overall rate of falling in the elderly, falls prevention programmes should then be tailored to the individual needs of the patient.

## Conclusions

It is well recognised that falls assessment and prevention programmes are multi-factorial. Evidence from this systematic review of diagnostic accuracy suggests that a single assessment tool like the TUG should not be used to identify community dwelling older adults at increased risk of falls.

## Competing interests

The authors declare that they have no competing interests.

## Authors' contributions

All authors were involved in the study conception and design. EB performed a systematic search of the literature. Both EB and RG screened potential articles, EB and FH evaluated the methodological quality of studies, RG, EB and CK acquired data for analysis, performed statistical analysis and interpretation of data and drafted the paper. TF critically revised the draft manuscript. All authors read and approved the final manuscript.

## Pre-publication history

The pre-publication history for this paper can be accessed here:

http://www.biomedcentral.com/1471-2318/14/14/prepub
